# Radiomic analysis in contrast-enhanced CT: predict treatment response to chemoradiotherapy in esophageal carcinoma

**DOI:** 10.18632/oncotarget.22304

**Published:** 2017-11-06

**Authors:** Zhen Hou, Wei Ren, Shuangshuang Li, Juan Liu, Yu Sun, Jing Yan, Suiren Wan

**Affiliations:** ^1^ State Key Laboratory of Bioelectronics, Laboratory for Medical Electronics, School of Biological Sciences and Medical Engineering, Southeast University, Nanjing, Jiangsu 210096, China; ^2^ The Comprehensive Cancer Centre of Drum Tower Hospital, Medical School of Nanjing University & Clinical Cancer Institute of Nanjing University, Nanjing, Jiangsu 210008, China

**Keywords:** esophageal carcinoma, computed tomography, radiomics analysis, predictor, treatment response

## Abstract

**Objectives:**

To investigate the capability of computed-tomography (CT) radiomic features to predict the therapeutic response of Esophageal Carcinoma (EC) to chemoradiotherapy (CRT).

**Methods:**

Pretreatment contrast-enhanced CT images of 49 EC patients (33 responders, 16 nonresponders) who received with CRT were retrospectively analyzed. The region of tumor was contoured by two radiologists. A total of 214 features were extracted from the tumor region. Kruskal-Wallis test and receiver operating characteristic (ROC) analysis were performed to evaluate the capability of each feature on treatment response classification. Support vector machine (SVM) and artificial neural network (ANN) algorithms were used to build models for prediction of the treatment response. The statistical difference between the performances of the models was assessed using McNemar’s test.

**Results:**

Radiomic-based classification showed significance in differentiating responders from nonresponders. Five features were found to discriminate nonresponders from responders (AUCs from 0.686 to 0.727). Considering these features, two features (Histogram2D_skewness: *P* = 0.015. Histogram2D_kurtosis: *P* = 0.039) were significant for differentiating SDs (stable disease) from PRs (partial response) and one feature (Histogram2D_skewness: *P* = 0.027) for differentiating SDs from CRs (complete response). Both classifiers showed potential in predicting the treatment response with higher accuracy (ANN: 0.972, SVM: 0.891). No statistically significant difference was observed in the performance of the two classifiers (*P* = 0.250).

**Conclusions:**

CT-based radiomic features can be used as imaging biomarkers to predict tumor response to CRT in EC patients.

## INTRODUCTION

Esophageal carcinoma is the eighth most commonly occurring types of malignancy, including more than 450.000 new cancer diagnoses yearly, and also the sixth leading cause of cancer-related mortality with an estimated approximately 400.000 deaths every year [1]. Most people are diagnosed with esophageal cancer present with locally advanced disease, to which concurrent chemoradiotherapy has emerged as a standard treatment [2]. However, locally advanced esophageal cancer has only 5-year overall survival of 36-47% after the CRT [3-5]. Therefore, a non-invasive prediction approach is expected to identify those who are at higher risk of poor response after CRT. Literatures showed that early selected patients with poor response to CRT may benefit from salvage surgery, with long-term survival [6, 7]. Therefore, identification of these patients prior to treatment would allow modification of their therapeutic plan and/or intensification of radiation dose to reduce unnecessary toxicity and improve prognosis.

Tumor internal microscopic differences (e.g., high cell density, necrosis, proliferation, hemorrhage, and hypoxia) are well-recognized features of malignancy that are related to worse prognosis, as well as to poorer response to treatment. Particularly, heterogeneity of the tumor blood supply will lead to the formation of cell hypoxia. The existence of hypoxic tumor cells increased tumor aggression and resistance to treatment [8] and is one of the most important reasons for distant metastasis [9]. Recent advances in radiomic analysis have been able to objectively and precisely quantify the tumor heterogeneity for predicting treatment response and prognosis.

Radiomic [10-12] is an emerging field that converts medical imaging to a set of high dimensional and quantitative features, including parameters not easily visible and quantifiable by simple visual analysis. By assessing the features of shape, texture, and transformation within a tumor lesion, radiomic analysis has the potential to provide complementary information relating to the tumor phenotype (e.g., shapes irregularity, heterogeneity or necrosis) [13-15]. Recently, many studies have shown that radiomic analysis could potentially provide a biomarker for the prediction of distant metastasis (DM) [16], treatment response [17-20], and radiation pneumonitis (RP) after radiotherapy [21, 22].

To our knowledge, there are few studies investigating the potential of radiomic analysis based on contrast-enhanced CT to predict treatment response in EC patients to CRT, particularly for the combination of multivariable prediction models, which may serve as an assistive tool for clinically accurate prediction. Therefore, the purpose of our study was to evaluate the power of radiomic features derived from pretreatment contrast-enhanced CT images combined with supervised machine-learning techniques in predicting therapeutic response to CRT in EC patients.

## RESULTS

### Treatment response after CRT

A total of 49 EC patients finished the treatment and observation. The evaluation of curative effect was performed 1 month after CRT. Patients classified as responders and nonresponders were 33 cases (17 CR, 16 PR) and 16 cases (16 SD, 0 PD), respectively.

### Feature inter-observer variability assessment

Five features extracted from two sets of contours delineated separately by two radiologists showed poor reproducibility (ICC < 0.8). In the other word, a total of 209 features were considered to be highly reproducible (ICC ≥ 0.8). The details were summarized in Table 1.

**Table 1:** ICC of features resulting from two radiologists contouring

Feature Type	ICC<0.8
Shape-based	0/4
Histogram-based	1/6
Texture-based	1/91
Transform-based	3/98

ICC, intra-class correlation coefficient.

### Predictive capabilities

**Figure 1:**
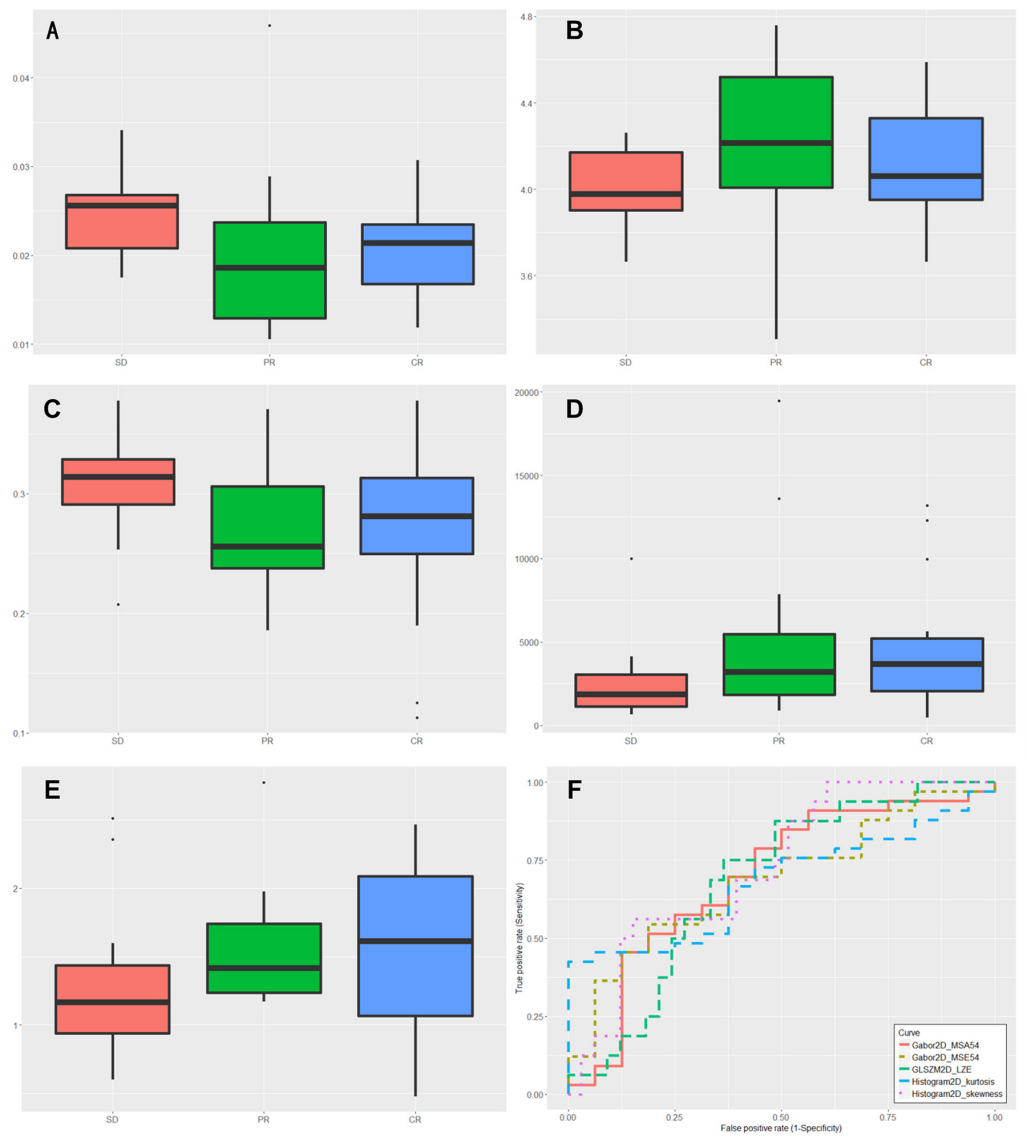
Box plots of the amplitudes of features, successfully differentiating nonresponders (stable disease [SD]) from responders (complete response [CR], partial response [PR]) **(A)** Histogram2D_skewness (*P*=0.0078). **(B)** Histogram2D_kurtosis (*P*=0.0355). **(C)** GLSZM2D_LZE (*P*=0.0396). **(D)** Gabor2D_MSA-54 (*P*=0.0418). **(E)** Gabor2D_MSE-54(*P*=0.0465). **(F)** ROC curve for Histogram2D_skewness, Histogram2D_kurtosis, GLSZM2D_LZE, Gabor2D_MSA-54 and Gabor2D_MSE-54 for classification responders from nonresponders.

The Kruskal-Wallis test was performed to all highly reproducible features with the results showing that 5 features could differentiate between responders (CRs and PRs) and nonresponders (SDs), 2 features (Histogram2D_skewness, *P* = 0.015; Histogram2D_kurtosis, *P* = 0.039) could differentiate between SDs and CRs, and one feature (Histogram2D_skewness, *P* = 0.027) could differentiate between SDs and CRs. The 5 features were Histogram2D_skewness, Histogram2D_kurtosis, GLSZM2D_LZE, Gabor2D_MSA-54, and Gabor2D_MSE-54, showed significantly different between responders and nonresponders. To discriminate between responders and nonresponders, we analyzed the Histogram2D_skewness with ROC curves and found a cut-off of 0.025, indicating that tumor lesions whose Histogram2D_skewness was higher than 0.025 were most likely from nonresponders (sensitivity = 56.25%, specificity = 84.85, AUC = 0.727; *P* = 0.007). Similar results were obtained from the ROC analysis of other 4 significant features. The detailed data were summarized in Table 2 and Table 3. The distribution of statistically significant parameters within different treatment response was shown in Figure 1. Additionally, for the pairwise comparison of AUCs between the 5 significant features, Delong’s test showed that there was a significant difference in predictive performance between Histogram2D_skewness and Histogram2D_kurtosis (AUCs: 0.727 vs. 0.689; *P* = 0.049).

**Table 2:** Features show statistical difference between responders and nonresponders

Feature	*P*-value	Standard Error	95%CI	AUC	Cut-off
Histogram2D_skewness	0.007	0.0743	0.581-0.845	0.727	>0.025
Histogram2D_kurtosis	0.035	0.0762	0.531-0.806	0.680	≤4.261
GLSZM2D_LZE	0.039	0.0777	0.537-0.811	0.686	>0.266
Gabor2D_MSA-54	0.041	0.0796	0.537-0.811	0.686	≤3066.039
Gabor2D_MSE-54	0.046	0.0865	0.547-0.818	0.695	≤1.200

AUC, area under the curve; CI, confidence interval; Responders, patients with CR and PR; Nonresponders, patients with SD.

**Table 3:** Features that classify different treatment responses

Feature type	Responders (CR, PR) Versus Nonresponders (SD)	SD Versus PR	SD Versus CR
Shape-based	None	None	None
Histogram-based	Histogram2D_skewness Histogram2D_kurtosis	Histogram2D_skewness Histogram2D_kurtosis	Histogram2D_skewness
Texture-based	GLSZM2D_LZE	None	None
Transform-based	Gabor2D_MSA-54 Gabor2D_MSE-54	None	None

CR, complete response; PR, partial response; SD, stable disease.

### Supervised classification and statistical comparison

Before model construction, feature reduction was performed to obtain the optimal parameter subsets on the basis of the wrapper-based method. With this method, our feature reduction process resulted in different optimal feature subsets for each model (SVM and ANN). The optimal feature subset for ANN contained 7 features and for SVM contained 9 features. Table 4 summarizes the optimal feature sets for each model in detail. Three features (Histogram2D_skewness, Gabor_MSA-42, and Gabor_MSA-55) were both selected in these 2 different feature sets and no shape-based features were selected. Table 5 summarizes the detailed results of the weighted average accuracy, precision, MCC, and AUC for each model. Results showed that both models have good performances in differentiating between responders and nonresponders, and higher accuracies were obtained (accuracy of ANN: 0.927; accuracy of SVM: 0.891).

**Table 4:** Optimal feature set obtained from wrapper-based feature selection

Feature type	SVM	ANN
Shape-based Histogram-based	None Histogram2D_skewness	None Histogram2D_skewness
Texture-based	GLCM3D_Correlation	GLCM3D_Entropy GLSZD3D_LZE GLSZD3D_SZHGE
Transform-based	Gabor_MSA-11, -22, -32, -37, -42, -44,-55	Gabor_MSA-42, -55 Gabor_MSE-26

SVM, support vector machine; ANN, artificial neural network.

**Table 5:** Summary of classification results obtained from training set using 10-Fold CV

Algorithm	TP rate	FP rate	Precision	Accuracy	F-Measure	MCC	AUC
ANN	0.973	0.064	0.974	0.972	0.973	0.936	0.927
SVM	0.892	0.256	0.906	0.891	0.884	0.743	0.818

SVM, support vector machine; ANN, artificial neural network; FP, false-positive; TP, true-positive; MCC, Matthews correlation coefficient.

Pairwise comparisons in McNemar’s test showed that there was no statistical difference between ANN and SVM models, indicating that the choice of the modeling algorithm was not of substantial importance (*P* = 0.250).

### Validation result

Table 6 shows the detailed results of the validation (testing set). ANN had higher accuracy than SVM (accuracy of ANN: 0.917; accuracy of SVM: 0.667), which was consistent with internal validation (training set).

**Table 6:** Classification results obtained from testing set

Algorithm	TP rate	FP rate	Precision	Accuracy	F-Measure	MCC	AUC
ANN	0.917	0.117	0.927	0.917	0.915	0.837	0.800
SVM	0.667	0.467	0.778	0.667	0.593	0.357	0.600

SVM, support vector machine; ANN, artificial neural network, FP, false-positive; TP, true-positive; MCC, Matthews correlation coefficient.

## DISCUSSION

Medical imaging is not only used for diagnosis and follow-up, but also image-based clinical parameters (e.g., TNM stage and ^18^F-FDG uptake) are used as predictors of treatment response. However, these widely used clinical indicators do not comprehensively capture the tumor phenotypic information. Radiomics method is able to quantify tumor phenotypical differences from medical images by analyzing a large number of imaging features that can be linked to clinical outcomes of the tumors. With this method, the quantified phenotypic information can be used as imaging biomarkers in response assessment of cancers.

Recently, large numbers of valuable imaging biomarkers of prognosis for patients with EC have been reported. For example, a previous study demonstrated the PET-based textures could be predictors of treatment response [20]. ROC curve analysis showed that tumor texture (i.e., homogeneity, entropy, and size zone variability) achieved higher AUCs (0.82 - 0.89) than any SUV measurement (AUCs from 0.59 to 0.70) in differentiating responders from nonresponders. Similarly, in their second study, several features (i.e., metabolic tumor volume [MTV], entropy, intensity variability, and zone percentage) were achieved good discriminatory power (AUCs from 0.80 to 0.90) for predicting nonresponders [23]. Based on baseline and post-treatment ^18^F-FDG PET scans, Tan et al. reported that changes in features over treatment appeared better predictive performance than pre or post-treatment assessment alone [24]. However, PET is expensive and time-consuming. In the long-term follow-up of cancer patients, CT is still the main imaging method which is performed in routine clinical practice. Ganeshan et al. [19] reported that CT-based textural heterogeneity has the potential to provide a prognostic indicator for survival. In their study, coarse uniformity (OR = 4.45; *P* = 0.039) showed the predictive power by Cox regression analysis. Yip et al. [25] explored the value of contrast-enhanced CT image features before and after neoadjuvant CRT and corresponding changes for the prediction of therapeutic response. The study suggested that two features (pre and post treatment SD) were found to be associated with treatment response. However, the aforementioned studies only used histogram-based and less texture-based features, which could not comprehensively assess the phenotype of the tumor. And no multi-parameter prediction model was established to dig out the predictive value of the multiple feature combination.

In present work, Shape-based and histogram-based metrics are global features that characterizing the geometric properties of the tumor and the overall statistical characteristics of the pixel gray values in tumor lesion, respectively. With these methods, Histogram2D_skewness and Histogram2D_kurtosis can be applied to distinguish nonresponders (SDs) from responders (CRs and PRs). Histogram2D_skewness and Histogram2D_kurtosis also have the same ability to discriminate PRs from SDs and Histogram2D_skewness can be used to discriminate CRs from SDs. The texture-based features depicted the spatial arrangement of the voxels and the change of local intensity in tumor region [26]. In the other words, the distribution of pixels in heterogeneous tumors showed more irregular than that in homogeneous tumors [22]. GLSZM2D_LZE, corresponding to the variability in the size and intensity of 2D ROIs, can be used to classify responders and nonresponders. Our radiomic model contained more features than the model constructed by Vallières et.al [27] by adding Gabor transform and LoG filter approach. Gabor transform is a form of short time Fourier transform that computing features via time-frequency analysis with different frequencies and orientations [28], and LoG filter is a differential operator applied to highlight the texture of different coarseness within an image first smoothened by the Gaussian filter according to the sigma value [29]. We showed the power of Gabor2D_MSA-54 and Gabor2D_MSE-54 to differentiate the nonresponders from responders. The results demonstrate the possibility of radiomic features extracted from pre-treatment CT images to differentiate different treatment response.

To our knowledge, multiple features combination can provide a comprehensive view to depict entire tumor, furthermore, achieve a full evaluation of their prognostic power. Zhang et al. [30] showed that features combined SVM model achieved a higher accuracy in predicting treatment response of ECs to CRT, whereas, there was no independent validation set (testing set) was used to evaluate the real performance of the model. In our study, SVM and ANN classifiers were performed on the training set (n = 37) and then validated on a testing set (n = 12). To minimize the risk of modeling over-fitting and bias, we used a robust processing approach: feature reproducibility assessment, wrapper-based feature selection, and model construction with 10-fold cross-validation. With these processes, the ANN model showed better performance than the SVM model, although the difference was not statistically significant. These predictive models may be of future interest as a clinical adjunct tool in response prediction. Overall, this demonstrates that radiomic models have the potential for predicting treatment response in patients with EC, however, this need to be further confirmed in larger prospective cohorts.

Literature and clinical experiences show pathology difference in esophageal carcinoma between Asian and Western populations [1]. Asian patients with esophageal carcinoma are mostly squamous cell carcinoma, and for the Western patients are mostly adenocarcinoma. By extrapolation of this different pathology of Esophageal carcinoma, treatment response [31] and radiation-induced complication [32] may be different between these two populations. Thus, the results from squamous EC in our study are inappropriate to the adenocarcinoma EC from the other population. In addition, our results may be moderated by several limitations in our study: small sample size and the retrospective nature of this study. These limitations may have an impact on the reliability of our result, keeping nevertheless the fact that our radiomic models predict significantly in treatment response.

In conclusion, combined with supervised machine-learning techniques, radiomic features derived from pretreatment contrast-enhanced CT scans could serve as an effective tool for the prediction of treatment response to CRT in EC patients, with the advantage of low cost, using existing CT image sets, without subjecting patients to further radiation exposure or imaging.

## MATERIALS AND METHODS

### Patient database

The retrospective database contained pretreatment contrast-enhanced CT scans from 49 patients who were histologically diagnosed as esophageal squamous cell carcinoma at Nanjing Drum Tower Hospital Cancer Center since March 2015 to December 2016. The patient characteristics were summarized in Table 7 and Table 8. All the patients were considered to be inoperable and not received chemotherapy or radiotherapy before CT scan. The other enrolled criteria included: normal cardiac, pulmonary, and hematologic function.

**Table 7:** Baseline characteristics of patients in training set

Characteristic	Responders (n=26)	Nonresponders (n=11)	*P* value
Age			
Median (range)	64(52-82)	66(56-81)	0.670^*^
Sex			
Male/ Female	15/11	7/4	>0.999^**^
TNM staging			
T1/T2/T3/T4	2/7/13/4	0/5/5/1	0.666^**^
N0/N1/N2	5/14/7	0/8/3	0.445^**^
M0/M1	25/1	10/1	0.512^**^
AJCC stage			
I/II/III/IV	1/12/12/1	0/4/6/1	0.737^**^

^*^Independent-samples *t*-test.

^**^chi-square test.

**Table 8:** Baseline characteristics of patients in testing set

Characteristic	Responders (n=7)	Nonresponders (n=5)	*P* value
Age			
Median (range)	56(50-61)	66(56-73)	0.198^*^
Sex			
Male/ Female	4/3	3/2	>0.999^**^
TNM staging			
T1/T2/T3/T4	0/3/3/1	0/3/1/1	0.773^**^
N0/N1/N2	0/4/3	0/3/2	>0.999^**^
M0/M1	7/0	4/1	0.417^**^
AJCC stage			
I/II/III/IV	0/3/4/0	0/1/3/1	0.735^**^

^*^Independent-samples *t*-test.

^**^chi-square test.

### Chemoradiotherapy

During the whole course of radiotherapy, patients underwent 2-3 cycles of synchronous chemotherapy (nedaplatin + docetaxel/paclitaxel). All patients’ primary tumors were irradiated with 2Gy per fraction in 30 fractions for intensity modulated radiation therapy (IMRT), and two patients received an additional dose (2Gy per fraction in 3 fractions) to the lymph node area in order to improve regional control. For all patients, the dose prescriptions were designed to cover at least 96% of planning target volume (PTV).

### Treatment evaluation

The treatment response was assessed one month after the treatment by CT image with contrast. Response Evaluation Criteria in Solid Tumors (RECIST) [33] was referred for evaluating the treatment responses. Complete response (CR), partial response (PR), stable disease (SD) and progressive disease (PD) were evaluated. Patients with CR or PR were considered responders, while patients with SD or PD were classified as nonresponders.

### CT image acquisition and tumor segmentation

**Figure 2:**
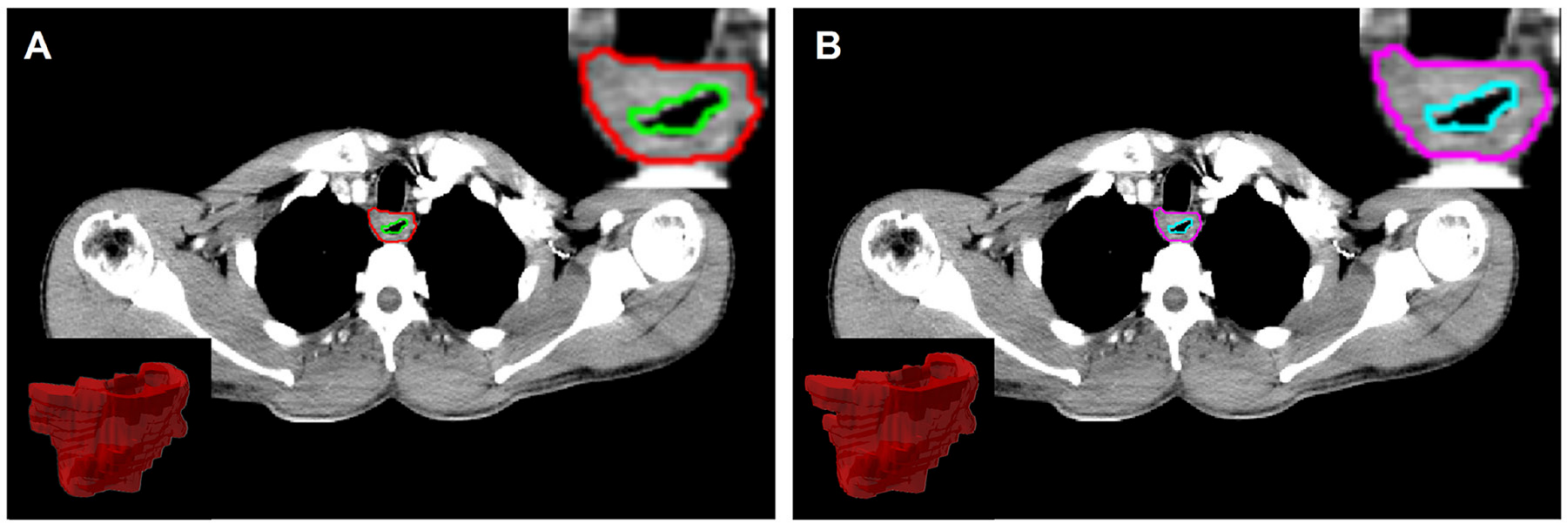
Region of interest (ROI) was contoured by two radiologists, and corresponding 2D/3D ROI (**A** for ROI-1 and **B** for ROI-2).

All planning CT scans were obtained from the same CT scanner (Philips Brilliance 6; Philips Healthcare, Best, the Netherlands) according to a standard clinical acquisition protocol (tube voltage, 120 kVp; tube current, 200 – 250 mAs; rotation time, 0.75 s ; pitch, 0.9; matrix, 512x512; field of view, 350 mmx350 mm; convolution kernel, standard), following intravenous injection of 300 mg/mL iodinated contrast agent at a rate of 3mL/s. In our database, the imaging slice thickness was 2.5mm or 3mm and the in-plane resolution was 0.97mm by 0.97mm. The primary 3D region of interests (ROIs) were manually delineated slice-by-slice in mediastinal window on Pinnacle software (Philips Medical Systems, Andover, MA) by two expert radiologist (Ren W. for ROI-1 and Li S. for ROI-2) and then reviewed by an experienced radiologist (Yan J.). For each ROI, the contours were drawn around the gross tumor volume (GTV) avoiding air, fat, and bone (Figure 2).

### Image preprocessing

Although patients underwent CT examination using the same scanner under a standard clinical acquisition protocol, changes of parameter settings may result in variation of intensity ranges. To minimize the effect of variations in image acquisition parameters and enhance the feature characteristics in quantitative image analysis, voxel values within the contoured ROIs were normalized with a finite gray-level range as follows:

$$V_{\left(x\right)}=\left[2^{k}\frac{I_{\left(x\right)}-min_{i\in \Omega }i}{max_{i\in \Omega }i-min_{i\in \Omega }i}\right]$$

Where 2^*k*^ represent the number of discrete values (16-128), I is the intensity of the raw image, and Ω is the set of pixels in the contoured ROI. This discretization process improves sensitivity relative to the raw data and weakens the image noise across all patient cohorts. Tixier et al. [20] showed that no statistically significant differences in the radiomic features derived using different resampling values (16, 32, 64, or 128). All subsequent reported results were obtained using 16 discrete values in the gray-level normalization process. All ROIs with voxel size of 0.97x0.97x2.5/3 *mm*^3^ were isotropically resampled to a voxel size of 1x1x1 *mm*^3^ using cubic interpolation algorithm to unify the voxel size across the cohort.

### Radiomic feature extraction

A feature computation module was developed for this study using MATLAB 2015a (Mathworks, Natick, MA, USA). DICOMs files (CT images + ROI structures) were first exported from Pinnacle software and then imported into above module to calculate radiomic features. In this study, 60 three-dimensional (3D) and 154 two-dimensional (2D) features were extracted. Specifically, three-dimensional texture features obtained from 3D ROIs base on density histogram, gray-level co-occurrence matrix (GLCM), gray-level run-length matrix (GLRLM), gray-level size zone matrix (GLSZM), and neighborhood gray-tone difference matrix (NGTDM) [27]. Two-dimensional texture features obtained from largest cross-sectional area of the tumor outline contained more metrics than 3D feature extractive method by adding gray-level gradient co-occurrence matrix (GLGCM), Laplacian of Gaussian (LoG) band-pass filters with different filter values (1.0 for highlighting fine texture, 2.0 for highlighting medium texture, 2.5 for highlighting coarse texture) and Gabor transform (5 scales, 8 orientation) [29, 34]. Conventional imaging features (shape-based) for lesion characteristics were also considered in this study. These shape-based metrics contained tumor volume, size (taken as the longest diameter of the 3D tumor lesion segmented from CT scans), solidity, and eccentricity [27].

In all, 214 radiomic features were extracted from four principal methods: shape-based (conventional metrics), histogram-based, texture-based (GLCM_2D/3D, GLRLM_2D/3D, GLSZM_2D/3D, NGTDM_2D/3D, GLGCM_2D, and LoG filter), and transform-based (Gabor transformation). For more detailed contents are summarized in the Supplementary Table 1.

### Statistical analysis

All statistical analyses were performed using R software version 3.3.2. Kruskal-Wallis test was used to compare the capability of each feature to differentiate patients (n = 49) with respect to treatment response after CRT. *P* < 0.05 was considered to be significantly different. Receiver operating characteristic (ROC) curve analysis was used to assess the performance of each studied features in distinguishing among various treatment responses (specificity, sensitivity, and 95% confidence intervals [CIs] were also calculated). Area under the curve (AUC) with a value of 1 indicates an ideal result, while values lower than 0.5 means insignificant. Delong’s test was performed to evaluate the statistical significance between AUCs of the influential features [35]. In addition, intra-class correlation coefficient (ICC) (“irr” package version 0.84 in R [36]) was used to quantify the feature reproducibility in repeat delineation. Radiomic features with ICC greater than 0.8 were considered as reproducible.

### Feature selection and model construction

Support vector machine (SVM) and artificial neural network (ANN) algorithms were performed to build models for the prediction of treatment response. The patients were separated into two groups: 37 patients (26 responders, 11 nonresponders) for training and 12 patients (7 responders, 5 nonresponders) for testing. The clinical information was summarized in Table 7 and Table 8. To avoid model overfitting and reduce the training time, the number of features should be reduced firstly. Based on the training group, wrapper-based feature selection method [37] was used to obtain an optimal feature subset for the specific model (SVM or ANN). It ranks all features by recursively removing features and then evaluating the predictive ability of the remaining features without missing any critical ones.

To evaluate classification performance, K-fold cross-validation (CV) method was served as the internal validation in the training set. The 10-fold CV was used, as the predictive performance had a good likelihood of closely reflecting the real performance with high efficiency [30]. True positives (TP), false positives (FP), true negatives (TN), and false negatives (FN) were obtained to calculate the sensitivity, specificity, and accuracy of the predictions. In addition, Matthews correlation coefficient (MCC) was used to measure prediction ability of the classifier. The MCC ranged from -1 to +1, values close to +1 represented ideal prediction, 0 indicated the equivalent of a random guess, and -1 implied the inverse prediction.

### Statistical comparison between ANN and SVM classifiers

McNemar’s test was performed to determine whether the predictive performance of different classifiers was significantly different [38]. The test was conducted on the outcomes achieved from the 10-fold CV.

### Validation

Patients (n = 12) who were not involved in classifier establishment were then served as a separate validation set. The confusion matrix containing prediction result was obtained from the established models to calculate the specificity and accuracy.

## SUPPLEMENTARY MATERIALS TABLE




