# Supplementary material for: Radiomic analysis in contrast-enhanced CT: predict treatment response to chemoradiotherapy in esophageal carcinoma
Source: Oncotarget. 2017 Nov 6;8(61):104444–54. doi: 10.18632/oncotarget.22304 (PMC5732818; doi:10.18632/oncotarget.22304)
Supplement: Supplementary file 1 [file oncotarget-08-104444-s001.pdf]

Radiomic analysis in contrast-enhanced CT: predict treatment response to chemoradiotherapy in esophageal carcinoma

SUPPLEMENTARY MATERIALS

Supplementary Table 1: Texture Type and Associated Features.

See Supplementary File 1
