# Supplementary material for: Radiomic analysis in contrast-enhanced CT: predict treatment response to chemoradiotherapy in esophageal carcinoma
Source: Oncotarget. 2017 Nov 6;8(61):104444–54. doi: 10.18632/oncotarget.22304 (PMC5732818; doi:10.18632/oncotarget.22304)
Supplement: Supplementary file 2 [file oncotarget-08-104444-s002.docx]

TABLE S1.Texture Type and Associated Features

| Feature type | Methods | Feature name |
| --- | --- | --- |
| Shape-based |  | Volume  Diameter_3D  Solidity  Eccentricity |
| Histogram-based | Histogram 3D/2D | Variance  Skewness  Kurtosis |
| Texture-based | GLCM 3D/2D | Energy |
|  | GLRLM 3D/2D  GLSZM 3D/2D  NGTDM 3D/2D  GLGCM 2D  LoG filter 2D  (filter widths: 1.0,fine;2.0,medium;2.5,coarse) | Contrast  Correlation  Homogeneity  Variance  Sum Average  Entropy  Short Run Emphasis (SRE)  Long Run Emphasis (LRE)  Gray-Level Non-uniformity (GLN)  Run-Length Non-uniformity (RLN)  Run Percentage (RP)  Low Gray-Level Run Emphasis (LGRE)  High Gray-Level Run Emphasis (HGRE)  Short Run Low Gray-Level Emphasis (SRLGE)  Short Run High Gray-Level Emphasis (SRHGE)  Long Run Low Gray-Level Emphasis (LRLGE)  Long Run High Gray-Level Emphasis (LRHGE)  Gray-Level Variance (GLV)  Run-Length Variance (RLV)  Small Zone Emphasis (SZE)  Large Zone Emphasis (LZE)  Gray-Level Non-uniformity (GLN)  Zone-Size Non-uniformity (ZSN)  Zone Percentage (ZP)  Low Gray-Level Zone Emphasis (LGZE)  High Gray-Level Zone Emphasis (HGZE)  Small Zone Low Gray-Level Emphasis (SZLGE)  Small Zone High Gray-Level Emphasis  (SZHGE)  Large Zone Low Gray-Level Emphasis (LZLGE)  Large Zone High Gray-Level Emphasis  (LZHGE)  Gray-Level Variance (GLV)  Zone-Size Variance (ZSV)  Coarseness  Contrast  Busyness  Complexity  Strength  Small Gradient Emphasis (SGE)  Large Gradient Emphasis (LGE)  Gray Inhomogeneous (GI)  Gradient Gray Inhomogeneous (GGI)  Gradient Energy  Mean Gray  Mean Gradient  Gray Variance  Gradient Variance  Mixture Entropy  Gray Entropy  Gradient Entropy  Gradient Inverse Difference Moment (GIDM)  Gradient Correlation  Gradient Difference Moment (GDM)  Mean intensity _fine/medium/coarse_  Entropy _fine/medium/coarse_  Uniformity _fine/medium/coarse_  Mean intensity _fine_/Mean intensity _medium_  Mean intensity _fine_/Mean intensity _coarse_  Mean intensity _medium_/Mean intensity _coarse_  Entropy _fine_/ Entropy _medium_  Entropy _fine_/ Entropy _coarse_  Entropy _medium_/ Entropy _coarse_  Uniformity _fine_/ Uniformity _medium_  Uniformity _fine_/ Uniformity _coarse_  Uniformity _medium_/ Uniformity _coarse_ |
| Transform-based | Gabor wavelet 2D  (5 scale, 8 orientation) | 40 mean square energy (MSE)  40 mean square amplitude (MSA) |

GLCM, Gray-level co-occurrence matrix; GLRLM, Gray-level run-length matrix; GLSZM, Gray-level size zone matrix; NGTDM, Neighborhood gray-tone difference matrix; GLGCM, Gray-level gradient co-occurrence matrix; LoG, Laplacian of Gaussian spatial band-pass filter; 3D, Methods performed on three-dimensional ROI; 2D, Methods performed on two-dimensional ROI with the largest cross-sectional area of the tumor outline.
